# COVID-19 and mental distress among health professionals in eight European countries during the third wave: a cross-sectional survey

**DOI:** 10.1038/s41598-024-72396-x

**Published:** 2024-09-12

**Authors:** Frieder Dechent, Gwendolyn Mayer, Svenja Hummel, Steffen Moritz, Charles Benoy, Rosa Almeida, Raquel Losada Durán, Oscar Ribeiro, Vincenza Frisardi, Ilaria Tarricone, Silvia Ferrari, Cédric Lemogne, Christian Huber, Steffi Weidt, Jobst-Hendrik Schultz

**Affiliations:** 1 Universität Basel, Universitäre Psychiatrische Kliniken (UPK) Basel, Basel, Switzerland; 2 Universitätsklinikum Heidelberg, Klinik für Allgemeine Innere Medizin und Psychosomatik, Heidelberg, Germany; 3 Universitätsklinikum Hamburg-Eppendorf, Klinik und Poliklinik für Psychiatrie und Psychotherapie, Hamburg, Germany; 4 Centre Hospitalier Neuro-Psychiatrique, Ettelbruck, Luxembourg; 5 Fundación INTRAS, Valladolid, Spain; 6 University of Aveiro, CINTESIS@RISE, Department of Education and Psychology, Aveiro, Portugal; 7 IRCCS Azienda Ospedaliero Universitaria di Bologna, Bologna, Italy; 8 University of Bologna, Bologna, Italy; 9 University of Modena and Reggio Emilia, Modena, Italy; 10 Université Paris Cité and Université Sorbonne Paris Nord, Inserm, INRAE, Center for Research in Epidemiology and StatisticS (CRESS), Paris, France; 11 Hôpital Hôtel-Dieu, Service de Psychiatrie de l’adulte, AP-HP, Paris, France; 12 Psychiatrie St. Gallen, St. Gallen, Switzerland

**Keywords:** COVID-19, Europe, Anxiety, Depression, Medical professionals, Mental health, Stress, Stressors, Public health, Health occupations

## Abstract

Even during the third wave of the COVID-19 pandemic health professionals were facing mental health challenges. The aim of this study was to examine the mental health of doctors, nurses and other professional groups in Europe and to identify differences between the professional groups. We conducted a cross-sectional online survey in 8 European countries. We asked for demographic data, whether the participants were exposed to COVID-19 at work, for main information sources about the pandemic, the Depression Anxiety Stress Scales (DASS-21), and major stressors. A MANCOVA was carried out to find predictors of mental health among health care professionals. The sample (N = 1398) consisted of 237 physicians, 459 nurses, and 351 other healthcare professionals and 351 non-medical professionals with no direct involvement in patient care. The mean mental health of all groups was affected to a mild degree. Major predictors for depression and anxiety were the profession group with higher scores especially in the group of the nurses and working directly with COVID-patients. In the third wave of the COVID-19 pandemic, the psychological burden on health professionals has remained high, with being nurse and working directly with COVID19 patients being particular risk factors for mental distress. We found as a main result that nurses scored significantly higher on depression and anxiety than practitioners.

## Introduction

### Background

After more than 3 years, the burden of disease in the general population due to the COVID-19 pandemic remains very high, with a total of over half a billion confirmed cases and more than 6 million deaths concerning COVID-19^1^. Calculations of disability-adjusted life years (DALYs) with a loss of 305,641 life years in Germany^2^ and 30,181 life years in Denmark^3^ even more emphasize the severe consequences of COVID-19 in the European population. Various protective measures such as lockdowns, social distancing regulations, or vaccinations were imposed to contain the medical consequences^4^, however an end of the Covid-19 impact is not in sight. This high burden of disease also places a particularly high burden on the health systems and, in association, on the mental health of health care professionals^5,6^. Although there is no compelling evidence that the impact of the pandemic on mental health was more pronounced in health care professionals than in the general population^7,8^, there is considerable heterogeneity in this matter and much remains to be studied with regards to the identification of specific risk factors in this population.

Much research so far has been done on the people who work in the health system and their mental distress. For example, in April 2020 numerous professionals in the health system in Spain showed symptoms of post-traumatic stress disorder, anxiety disorders, and depression, with women and younger people showing an increased risk^9^. A Portuguese study among physicians in 2020 showed that working directly with patients with COVID-19 also led to more symptoms of stress, depression, and anxiety, with female physicians being particularly affected^10^. A follow-up study over a year showed a significant decrease in stress and depression values, but the authors found a prospective connection between depression, stress, and symptoms of a post-traumatic stress disorder. Moreover, the female gender, but also the fear of being infected or infecting people close to them, and the reported insufficient access to protective material were identified as risk factors^11^. A higher burden on nurses in comparison to the physicians could be demonstrated in multiple studies^9,12–15^.

In addition to the professional group affiliation, direct contact with patients with COVID-19 and the respective medical ward also seem to play a role^16^. The workload in intensive care units in particular has been observed to directly increase mental health symptoms of the employees. This was particularly the case in England, where the number of ICU staff with mental health symptoms was high in younger nurses and fluctuated depending on the severity of the second wave^17^. In a study in Switzerland working in intensive care units was described with increased symptoms of anxiety and depression^18^.

Investigating factors causing distress during the pandemic, the role of information and media consumption needs special consideration. Even before the outbreak of the COVID-19 pandemic social media consumption played a major role in mental well-being. A study showed that the emotions expressed by others on social media have an impact on the emotions of the user^19^. Current studies about the impact of social media use during the COVID-19 pandemic have also shown an elevated mental burden on people who increasingly obtain their information in social networks^20,21^ and even chronic stress and panic have been observed due to the so called “infodemic” that comprises the fact that misinformation have been spread as a kind of “digital epidemic”^22^. However, little is known about the potential role of social media exposure on psychological distress among healthcare professionals.

### Objectives of the study

This study aims at understanding the mental health and its conditions of physicians, nurses, other medical staff and non-medical professionals in the health care system of 8 European countries during the third wave of COVID-19 with increased sanitary measures in Europe.

We aimed to focus on risk factors either specific to health-care professionals (e.g., working in ICU) or non-specific but overlooked so far in this specific population (e.g., exposure to social media).

A key focus was put on the professional groups of physicians and nurses with regard to the severity of symptoms in their respective ward, stressors, working hours, and sources of COVID-related information.

## Methods

### Study design and procedures

A cross-sectional survey was carried out by means of an online questionnaire that has been developed for the purpose of this and two former studies, that were carried out in 2020^23,24^. The questionnaire was made available on SoSciSurvey^25^ and distributed within Europe in six languages (English, French, German, Italian, Portuguese, and Spanish). The link to the survey was distributed via email to personal and professional networks following a snowball sampling approach. Invitation emails were sent to colleagues at affiliations of all co-authors, i.e. clinical and research institutions in, France, Germany, Italy, Luxembourg, Portugal, Spain and Switzerland. It was then further distributed to related institutions and to partner organizations, hospitals, and professional associations. Participants were also recruited via personal networks or public social networking groups, such as medical or nursing groups at Twitter, LinkedIn, and Facebook. The survey was launched on 25th November 2021 and closed on 28th February 2022.

The qualitative results of this survey that included answers in open text fields in this study period and those of the former study period were published elsewhere^26^.

We follow the reporting guidelines of the STROBE statements for observational studies^27^.

### Ethical considerations

The study was performed in accordance with the Declaration of Helsinki and was approved by the ethics committee of the Heidelberg University Medical Faculty (S-361/2020). Data collection was organized in compliance with the European General Data Protection Regulation (GDPR). The survey questionnaire was distributed in 8 European countries and all healthcare workers and associated staff at hospitals as well as non-medical staff were eligible to participate. Informed consent was obtained from the participants online prior to participation. No allowance was given for participating in the survey. All questionnaires were completed anonymously. Data security was granted by use of the SSL-encrypted platform SoSci Survey^25^.

### Measures

The questionnaire has been described in detail in a former publication^24^. However, as some parts have been changed due to new requirements, the structure of the questionnaire will be repeated and the new sections described: in the first part, we asked for demographic data, exposure to people infected with COVID-19 in their daily life and at work, working hours per day, and the means by which people gain information about COVID-19 (multiple choice: Internet, social media, TV, communication with colleagues, other). Similar to the previous study^24^ we asked the mental health status using the short version of the Depression-Anxiety Stress Scales (DASS-21)^24,28,29^ and for stressors of nurses and physicians that has been derived from a previous study during SARS epidemic in 2003^29^. The DASS questionnaire consists of 21 questions, seven each of which belong to the depression, anxiety, and stress subscale. Responses are given on a 4-point Likert scale ranging from “did not apply to me at all” = 0, to “applied to me very much or most of the time” = 3. 

### Data analysis

The data was analyzed with IBM SPSS statistics 26^30^. We collected data of 1439 participants and removed all datasets that were filled out outside the targeted countries (n = 41). The final analysis comprised data of 1398 participants. Missing values were omitted from the calculations without replacement.

We calculated descriptive statistics and reported frequencies, means, standard deviations, and percentages.

Following the manual of the DASS-21^28^, individual sum scores were calculated based on the depression, anxiety and stress subscales and multiplied by two. The depression subscale score was categorized as normal (0–9), mild (10–13), moderate (14–20), severe (21–27), and very severe depression (28+). The anxiety subscale score was categorized as normal (0–7), mild (8–9), moderate (10–14), severe (15–19), and extremely severe anxiety (20+). The total stress subscale score was categorized as normal (0–14), mild (15–18), moderate (19–25), severe (26–33), and extremely severe stress (34+)^5^. These subscales were then grouped as normal/mild; moderate; severe/very severe, following our previous approaches to ease the interpretation^23,24^, as mild symptoms of mental disorders show a high prevalence regardless of a pandemic like COVID-19^31^.

We created four groups: (1) physicians (including physicians and dentists), (2) nurses, (3) other health care professionals, which included “other job in healthcare system” and “volunteer in the context of medical pandemic aid”, and (4) non-medical staff consisting of professionals who usually do not work directly with the patients or their immediate environment.

A multivariate analysis of covariance (MANCOVA) was computed for each of the three DASS-21 scores as the dependent variable, with the two predictors being the profession group and contact with people infected by COVID at work, and working hours per week, own infection with COVID, country where the participant is actually living, age and gender as a covariate. We chose a robust test statistic of Pillai in case of violation of assumptions of normality and homogeneity of covariance matrices^32^.

We then focused on the groups of physicians and nurses only for whom we reported the three DASS-21 scores according to medical departments, as well as major stressors. A t-test was carried out for differences. Major ways of gaining information on COVID were calculated as frequencies and percentages. On the basis of this data, we carried out chi-square tests to find differences between the two groups. Finally, Eta-coefficients (η) were calculated to show associations between DASS-21 scores and the use of information sources.

In all analyses, *p* values < 0.05 were considered statistically significant.

## Results

### Participants

The sample size was 1398 people, of whom 237 were physicians, 459 were nurses, 351 comprised other healthcare professionals and 351 were non-medical professionals. The group of other health professionals consisted mainly of psychologists, educators, laboratory technicians, occupational therapists, dance and movement therapists, pharmacists, and medical-technical assistants. The group of non-medical professionals was very heterogeneous. This group consisted mainly of administrative employees, secretaries, researchers, educators, and computer scientists. The ages of the participants ranged from 19 to 78 (median: 42 years). A total of 369 (26.4%) males, 1024 (73.2%) females, and 5 non-binary people (0.4%) took part in the survey.

The distribution of people across countries is shown in Table 1. The distribution of the professional groups in the different countries are provided in the Supplementary File 1.

**Table 1:** Demographic characteristics of the study participants from November 2021 to February 2022 in 8 European countries.

Characteristics	Participants, n (%)
Age (years), mean (SD)	42.22 (10.6)
Gender	
Male	369 (26.4)
Female	1024 (73.2)
Non-binary	5 (0.4)
Country	
Belgium	93 (6.7)
France	322 (23.0)
Germany	243 (17.4)
Italy	120 (8.6)
Luxemburg	348 (24.9)
Portugal	44 (3.1)
Spain	44 (3.1)
Switzerland	184 (13.2)
What is the population of the city you are living?	
Less than 5000 inhabitants	375 (26.8)
Between 5000 and 20,000 inhabitants	400 (28.6)
Between 20,000 and 100,000 inhabitants	259 (18.5)
Between 100,000 and 500,000 inhabitants	273 (19.5)
More than 500,000 inhabitants	91 (6.5)
Education	
Secondary education	97 (6.9)
Post-secondary non-tertiary education	331 (23.7)
First stage of tertiary education	793 (56.7)
Second stage of tertiary education	173 (12.4)
Primary or lower	4 (0.3)
Marital Status	
Single	233 (16.7)
Married	644 (46.1)
Divorced	102 (7.3)
Widowed	7 (0.5)
In relationship	393 (28.1)
Other	19 (1.4)
Children	
Yes	841 (60.2)
No	557 (39.8)
Profession	
Physician	234 (16.7)
Dentist	3 (0.2)
Nurse	459 (32.8)
Other healthcare professionals	351 (25.1)
Non-medical professionals	351 (25.1)
Total	1398 (100)

### Vaccination status and working conditions during COVID-19 during the third wave of the COVID-19 pandemic

Results on the share of participants with infection, vaccination, contact to COVID at work, working hours and the respective medical unit are displayed in Table 2.

**Table 2:** Infection, vaccination, contact to COVID at work, working hours and medical unit (frequencies and percentages).

Characteristics	Participants n (%)
Are/were you infected with COVID-19?	
Yes	295 (21.1)
No	1085 (77.6)
Have you already received a COVID-19 vaccine?	
Yes, I have received the first dose of the COVID-19 vaccine	36 (2.6)
Yes, I have received two doses on the COVID-19 vaccine	390 (27.9)
No, I have not received a COVID-19 vaccine	108 (7.7)
Yes, I have received three doses on the COVID-19 vaccine	864 (61.8)
Do you have contact at work with people infected with COVID-19?	
Yes	1027 (73.5)
No	371 (26.5)
Working hours per week	
< 20 h	32 (2.3)
20–30 h	78 (5.6)
30–40 h	298 (21.3)
40–50 h	506 (36.2)
50–60 h	268 (19.2)
60–70 h	136 (9.7)
> 70 h	80 (5.7)
In which medical unit do you work? (only for medical staff)	
ICU	99 (7.1)
General inpatient department	316 (22.6)
Outpatient department	98 (7.0)
Emergency department	53 (3.8)
Doctor’s office	42 (3.0)
Ambulant care	528 (37.8)
Other	87 (6.2)
Total	1223 (87.5)
Missing	175 (12.5)
Total	1398 (100)

### Opportunity to work from home (“home office”)

In Table 3 professional groups are presented who had the opportunity to work remotely.

**Table 3:** Professional groups who reported to be able to work remotely from November 2021 to February 2022 in 8 European countries.

Profession	Participants, n (%)
Physician	39 (16.5)
Nurse	32 (7.0)
Other healthcare professionals	73 (20.8)
Non-medical professionals	175 (49.9)
Total	319 (22.8)

### Mental Health (DASS-21)

Across all professions and countries, a share of 23.3% (n = 326) report levels of depression that can be categorized as a severe/very severe degree. A share of 18.2% (n = 255) express severe/very severe levels of anxiety and 25.4% (n = 355) voice severe/very severe levels of stress. Nurses and non-medical staff show the highest degree of burden in all three symptom profiles. More details are presented in Table 4. Details of DASS-21 scores in the different countries are provided in the Supplementary File 2.

**Table 4:** Results for the Depression Anxiety and Stress Scales-21 (DASS-21) for doctors, nurses, Other healthcare professionals and non-medical professionals from November 2021 to February 2022 in 8 European countries (means, standard deviations, frequencies and percentages).

	Participants, n	Mean (SD)	Normal/mild, n (%)	Moderate, n (%)	Severe/very severe, n (%)
DASS-21 depression					
Physicians	237	10.71 (11.03)	160 (67.5)	33 (13.9)	44 (18.6)
Nurses	459	13.14 (11.09)	261 (56.9)	85 (18.5)	113 (24.6)
Other healthcare professionals	351	12.17 (11.21)	212 (60.4)	60 (17.1)	79 (22.5)
Non-medical professionals	351	13.14 (11.21)	200 (57.0)	61 (17.4)	90 (25.6)
Total	1398	12.48 (11.10)	833 (59.6)	239 (17.1)	326 (23.3)
DASS-21 anxiety					
Physicians	237	5.42 (7.36)	186 (78.5)	29 (12.2)	22 (9.3)
Nurses	459	8.55 (8.87)	294 (64.1)	64 (13.9)	101 (22.0)
Other healthcare professionals	351	7.72 (8.44)	236 (67.2)	55 (15.7)	60 (17.1)
Non-medical professionals	351	8.86 (9.63)	214 (61.0)	65 (18.5)	72 (20.5)
Total	1398	7.89 (8.80)	930 (66.5)	213 (15.2)	255 (18.2)
DASS-21 stress					
Physicians	237	16.15 (11.01)	149 (62.9)	35 (14.8)	53 (22.4)
Nurses	459	17.45 (11.18)	272 (59.3)	67 (14.6)	120 (26.1)
Other healthcare professionals	351	16.93 (11.08)	212 (60.4)	56 (16.0)	83 (23.6)
Non-medical professionals	351	17.56 (11.81)	196 (55.8)	56 (16.0)	99 (28.2)
Total	1398	17.13 (11.29)	829 (59.3)	214 (15.3)	355 (25.4)

### Comparison of the mental health of physicians, nurses, other healthcare professionals and non-medical professionals and the professionals having contact with COVID patients versus having no contact

A statistical analysis with a MANCOVA with the working hours per week, possibility to work from home (“home office”), own infection with COVID, country where the participant is actually living, age and gender showed a significant effect for profession (Pillai trace = 0.016, F_3000_ = 2479, *P* = 0.008) and also for the fact of being in contact with COVID-19 patients or not (Pillai trace = 0.007, F_3000_ = 3432, *P* = 0.016). The covariates showed also significant results (Working hours (Pillai trace = 0.030, F_3000_ = 14,163, *P* < 0.001); own infection with COVID-19 (Pillai trace = 0.009, F_3000_ = 4361, *P* = 0.005); country (Pillai trace = 0.013, F_3000_ = 5916, P < 0.001); age (Pillai trace = 0.017, F_3000_ = 7788, *P* < 0.001); gender (Pillai trace = 0.021, F_3000_ = 9843, *P* < 0.001).

In the between-subject analysis for profession in two of three DASS-scores showed significant results (DASS-D *P* < 0.023, DASS-A *P* < 0.001, DASS-S *P* = 0.308). Physicians showed in all three DASS-scores the lowest scores (Table 4). For the people having contact at work with COVID-patients all three DASS-scores showed a significant difference (DASS-D *P* = 0.001, DASS-A = 0.032, DASS-S *P* = 0.016).

### Mental health in the medical units and workload of physicians and nurses

Depending on the workplace, the highest values on all three scales were found among staff in the intensive care units, ahead of staff in the general medical units and the emergency units (Table 5).

**Table 5:** Results for the Depression Anxiety and Stress Scales-21 (DASS-21) of physicians and nurses in the medical units (N = 696) from November 2021 to February 2022 in 8 European countries (means and standard deviations).

	N*	Dass-21 Depression M (SD)	Dass-21 Anxiety M (SD)	Dass-21 Stress M (SD)
ICU	85	15.04 (11.40)	9.95 (9.28)	20.21 (11.65)
General inpatient department	231	12.00 (10.84)	6.97 (8.54)	16.53 (10.43)
Outpatient department	61	11.44 (11.41)	6.69 (7.57)	15.54 (10.83)
Emergency department	43	10.23 (9.85)	5.67 (6.82)	15.91 (9.10)
Physician's office	38	9.95 (11.68)	4.74 (7.95)	13.79 (11.70)
Ambulant care	165	11.60 (11.05)	7.58 (8.66)	16.40 (11.84)
Other medical departments	64	14.59 (10.95)	8.88 (7.92)	19.78 (10.97)
Total/mean	687	12.25 (11.06)	7.43 (8.47)	16.98 (11.11)

*Missing: n = 9.

Over all four groups the correlation of working hours and DASS-21 Depression was significant with r (1396) = − .09 (p* = 0.001), while DASS-21 Anxiety was r(1396) = .03 (*p* = 0.35), and Dass-21 Stress was r (1396) = .14 (p* < 0.001).

### Job-related stressors of physicians and nurses

Among the medical staff, “uncertainty about when the epidemic will be under control” was rated highest, followed by “worry about inflicting COVID-19 on family”, “worry about lack of manpower”, “frequent modification of infection control procedures”, and “coworkers being emotionally unstable”. Participants were less concerned about to get blamed by their commanding officers. An overview of all stressors in the order of reported severity can be found in Table 6. Overall, all stressors were rated to a higher level by nurses than by physicians.

**Table 6:** Stressors for physicians (n = 237) and nurses (n = 459) during COVID-19 from November 2021 to February 2022 in 8 European countries (means and standard deviations).

Items^a^	Physicians mean (SD)	Nurses mean (SD)	All mean (SD)	*P*
Uncertainty about when the epidemic will be under control	1.86 (0.95)	2.20 (0.85)	2.08 (0.90)	< 0.001
Worry about inflicting COVID-19 on family	1.74 (1.09)	2.16 (0.96)	2.02 (1.02)	< 0.001
Worry about lack of manpower	1.73 (1.07)	2.09 (1.00)	1.97 (1.04)	< 0.001
Frequent modification of infection control procedures	1.55 (0.92)	1.83 (0.97)	1.73 (0.96)	< 0.001
Coworkers being emotionally unstable	1.30 (0.98)	1.74 (0.99)	1.59 (1.00)	< 0.001
Patients’ emotional reaction	1.21 (0.93)	1.69 (0.99)	1.53 (0.99)	< 0.001
Deterioration of patients’ condition	1.21 (0.97)	1.68 (1.03)	1.52 (1.04)	< 0.001
Protective gears cause physical discomfort	1.24 (0.96)	1.64 (1.09)	1.50 (1.06)	< 0.001
Patient families’ emotional reaction	1.12 (0.96)	1.60 (1.00)	1.44 (1.01)	< 0.001
Worry about nosocomial (= intra-hospital) spread	1.30 (0.96)	1.47 (1.03)	1.41 (1.01)	0.036
Worry about getting infected	1.21 (0.94)	1.52 (1.01)	1.41 (1.00)	< 0.001
Conflict between duty and safety	1.00 (0.95)	1.45 (0.99)	1.30 (0.98)	< 0.001
Being without properly fitted environment	1.07 (1.02)	1.37 (1.06)	1.27 (1.06)	< 0.001
Documentation and reporting procedures unclear	1.06 (0.98)	1.37 (1.00)	1.26 (1.00)	< 0.001
Worry about being negligent and endangering patients	1.11 (0.99)	1.32 (1.07)	1.25 (1.04)	0.005
Worry about lack of proper knowledge and equipment	1.00 (0.93)	1.26 (1.00)	1.17 (0.99)	0.001
Protective gears being a drag in providing quality care	0.86 (0.98)	1.29 (1.04)	1.15 (1.04)	< 0.001
Be infected by the colleagues	0.87 (0.88)	1.22 (1.00)	1.10 (0.97)	< 0.001
Coworkers displaying COVID-19-like symptoms	0.76 (0.82)	1.26 (0.99)	1.09 (0.96)	< 0.001
Equivocal definition of the responsibility between doctors and nurses	0.67 (0.88)	1.29 (1.03)	1.08 (1.02)	< 0.001
Yourself displaying COVID-19-like symptoms	0.78 (0.87)	1.19 (1.00)	1.05 (0.98)	< 0.001
Worry about being negligent and endangering coworkers	0.84 (0.87)	1.05 (1.02)	0.98 (0.97)	< 0.001
Blaming from commanding officers	0.49 (0.81)	1.04 (1.10)	0.85 (1.04)	< 0.001

^a^Responses to the question: “When you think about COVID-19 in your life and work, how often did you think or worry about the following things?” (0 = not at all, 3 = very much).

### Information on COVID-19

In Table 7, we see the different sources of information. Both television and social media were less frequently reported by physicians as being a main source of information about COVID-19 (*P* < 0.001).

**Table 7:** Answers to the question “How do you mainly inform yourself about COVID-19?” given by physicians and nurses from November 2021 to February 2022 in 8 European countries (frequencies and percentages or respective n; multiple choice possible).

Medium	Participants, n (%)		
	Physicians	Nurses	Total
Newspaper	92 (38.8)	230 (50.1)	322 (46.3)
TV	73 (30.8)	268 (58.4)	341 (49.0)
Social media	31 (13.1)	142 (30.9)	173 (24.9)
Internet	165 (69.6)	276 (60.1)	441 (63.4)
Communication with colleagues	145 (61.2)	305 (66.4)	460 (64.7)
Other	54 (22.8)	66 (14.4)	120 (17.3)

A further analysis revealed a weak positive association in the depression, stress, and anxiety scales with the use of social media as a main source of information about COVID-19 (DASS-D *η* = 0.05; DASS-A* η* = 0.11; DASS-S *η* = 0.88). More associations could be shown for television and internet, but only in the case of anxiety (DASS-A *η* = 0.05 and *η* = 0.06 respectively).

## Discussion

### Principal findings

One of our main findings in this European wide study is that nurses and other medical and non-medical health workers had higher scores in depression and anxiety scores as measured by the DASS-21 in comparison to physicians. The study was carried out to ask for symptoms and predictors of the mental health of physicians, nurses, and other professions in and outside of direct patient care during the third wave of the pandemic in winter 2021/2022. In the analysis, we considered frequently described risk factors such as age, gender, workload and country of residence of the participants. In total, the proportion of nurses with moderate and severe depression was higher than that of physicians. For anxiety, too, the proportion of nurses in the moderate and severe categories was higher. Similar findings were also reported in different studies and reviews^12,15,33,34^. In a Belgian sample, there was no direct influence of whether someone works directly with COVID patients, but rather an influence of the professional group on burnout and anxiety symptoms as well as on insomnia^13^. A study of Italian health professionals showed that nurses suffered more from overall psychological distress than physicians^14^. This difference was also demonstrated in a Belgian study^35^. An Italian study^36^ revealed a significantly higher risk for nurses and explained this, among other causes, by the fact that nurses were less involved in the decision-making processes and also spent significantly more time directly exposed to the infectious patients during their work.

Another main finding of our study was the correlation between the stress and depression scores and the working hours. This association can be supported by previous studies^37^. A Dutch investigation was able to show a connection between an increase in the prevalence of burnout, the occurrence of COVID-19, direct contact with COVID-19 patients, and the hours worked by professionals in ICUs^38^.

Physicians and nurses who worked directly with COVID-19 patients also had higher values in all DASS scores in our study. This correlation is also described in other studies^10,39^. A similar association was found in an Australian sample, for example, where caregivers with direct contact with COVID-19 patients had the most pronounced emotional exhaustion, while non-medical professionals with no contact had the lowest scores^34^. A meta-analysis showed higher values for anxiety and depression in the group of professionals with contact to COVID-19 patients^40^. In this meta-analysis, women, married individuals, individuals with children, and nurses had relatively high scores in both depression and anxiety. In summary, in addition to one's own exposure, fear for family members seems to play an important role through the fear of infecting them through one’s own exposure. Other fears referred to staff shortage as well as the often-increased contact with seriously ill patients or the concern about the deterioration of their condition. Compared to the previous first-wave study by Hummel et al.^24^ who used a comparable design, similar overall values were shown in the individual DASS scores, although it must of course be added that this is not a follow-up study.

The most frequently mentioned stressors were “Uncertainty about when the epidemic will be under control” and “Worry about inflicting COVID-19 on family”, which were the same as in the previous study^24^. Especially the worry about infecting the family which was also described before^41^. For the stressors, the nurses consistently showed higher mean values than did the physicians, which basically fits with the mental burden of the nurses described above. This might be explained by the longer duration they have to work directly with the patients. In addition to the other stressors, deficits in the acquisition of knowledge are weighted significantly higher by the nurses than by the practitioners. Other results of our survey that included as well coping strategies, have been published elsewhere^26^. There, the acquisition of knowledge was also a successful coping strategy. There still seems to be a stressor in the third wave of the pandemic, although this problem was already recognized at the beginning of the pandemic^42^. This result is also comparable to former findings that describe even a worsening of mental distress but in non-comparable time periods^43–45^.

Another important result of our study was the correlation of the use of social media with higher values in all DASS scales, although this connection could not be seen for other sources of information. A possible explanation might argue that increased social media use and the associated increased exposure to corresponding content were associated with increased anxiety^20,21^. Interestingly, this connection has already been revealed by an experimental study before the pandemic^19^, where the reduction of positive expressions led to fewer positive and more negative posts, and the reduction of negative posts led to opposite findings. On the other hand, loneliness, isolation, workload and fears might increase the use of social media as a means of compensation especially in the case of nurses who were burdened to a certain extent^46^. We found a weak positive correlation of social media use and DASS-scores. In line with the literature this should be interpreted as an indication why increased use of social media is associated with more stress and the associated increased values in the DASS scores in our study. Whether more stress leads to increased use of social media or a higher frequency of social media use evokes higher stress levels cannot be derived from our study. Future investigations are necessary to come to a causal conclusion regarding mental health and social media use in specific professional groups.

### Limitations

Since the link to the online survey was distributed on the one hand through personal contacts and on the other hand via social networks, there was a very unequal distribution of the professional groups and of the gender of the participants in the different countries. In addition, non-occupational factors, such as the strictness of measures and the different dynamics of the pandemic in the individual countries over the survey period, certainly also played a role in mental health. We neither asked for details of institutional and governmental measures nor for the willingness of the participants to follow them. Overall, the participants cannot all be assigned to medical specialties or work areas, so that there could be an overrepresentation of employees in psychiatry. When asked about the sources of information, specific scientific journals were not asked about as a source of information for the participants. This can lead to a bias in the results, since a significant group of people and possibly a possible difference between the professional groups were not shown.

## Conclusion

In summary, our study continued to provide numerous indications that the COVID-19 pandemic still is a significant stress factor for the healthcare system. When scores for stress were not statistically different, we found as a main result that nurses scored significantly higher on depression and anxiety than practitioners. These differences were also reflected in the different levels of stressors that we evaluated for nurses and physicians. In addition to the positive correlation of working hours with stress and depression and the positive connection between direct contact with COVID-19 patients and increased anxiety, depression, and stress, the highest psychological burden was shown in employees of intensive care units. As a secondary result we found a weak positive correlation between social media use and the DASS-scores. Further investigations are needed to clarify the role of social digital media as negative *influencer* on the mental health of healthcare professionals.

## Supplementary Information

Supplementary Table 1.

Supplementary Table 2.

## Data Availability

The datasets generated during and/or analyzed during the current study are available from the corresponding author on reasonable request.
