# Publisher Correction: COVID-19 and mental distress among health professionals in eight European countries during the third wave: a cross-sectional survey

**DOI:** 10.1038/s41598-024-84239-w

**Published:** 2025-01-08

**Authors:** Frieder Dechent, Gwendolyn Mayer, Svenja Hummel, Steffen Moritz, Charles Benoy, Rosa Almeida, Raquel Losada Durán, Oscar Ribeiro, Vincenza Frisardi, Ilaria Tarricone, Silvia Ferrari, Cédric Lemogne, Christian Huber, Steffi Weidt, Jobst-Hendrik Schultz

**Affiliations:** 1 Universität Basel, Universitäre Psychiatrische Kliniken (UPK) Basel, Basel, Switzerland; 2 Universitätsklinikum Heidelberg, Klinik für Allgemeine Innere Medizin und Psychosomatik, Heidelberg, Germany; 3 Universitätsklinikum Hamburg-Eppendorf, Klinik und Poliklinik für Psychiatrie und Psychotherapie, Hamburg, Germany; 4 Centre Hospitalier Neuro-Psychiatrique, Ettelbruck, Luxembourg; 5 Fundación INTRAS, Valladolid, Spain; 6 University of Aveiro, CINTESIS@RISE, Department of Education and Psychology, Aveiro, Portugal; 7 IRCCS Azienda Ospedaliero Universitaria di Bologna, Bologna, Italy; 8 University of Bologna, Bologna, Italy; 9 University of Modena and Reggio Emilia, Modena, Italy; 10 Université Paris Cité and Université Sorbonne Paris Nord, Inserm, INRAE, Center for Research in Epidemiology and StatisticS (CRESS), Paris, France; 11 Hôpital Hôtel-Dieu, Service de Psychiatrie de l’adulte, AP-HP, Paris, France; 12 Psychiatrie St. Gallen, St. Gallen, Switzerland

Correction to: *Scientific Reports* 10.1038/s41598-024-72396-x, published online 12 September 2024

The original version of this Article contained an error in the spelling of the author Steffen Moritz which was incorrectly given as Moritz Steffen.

The original Article has been corrected.

