# Supplementary material for: COVID-19 and mental distress among health professionals in eight European countries during the third wave: a cross-sectional survey
Source: Sci Rep. 2024 Sep 12;14:21333. doi: 10.1038/s41598-024-72396-x (PMC11393084; doi:10.1038/s41598-024-72396-x)
Supplement: Supplementary file 1 — Supplementary Table 1. [file 41598_2024_72396_MOESM1_ESM.docx]

**Supplementary file 1: Distribution of the professional groups in the different countries from November 2021 to February 2022 (frequencies and percentages)**

|  | **Physicians n (%)** | **Nurses n (%)** | **Other healthcare professionals**  **n (%)** | **Non-medical professionals n (%)** | **Total  N (%)** |
| --- | --- | --- | --- | --- | --- |
| **Germany** | 32 (13.2) | 82 (33.7) | 60 (24.7) | 69 (28.4) | 243 (100.0) |
| **Belgium** | 2 (2.2) | 43 (46.2) | 26 (28.0) | 22 (23.7) | 93 (100.0) |
| **Switzerland** | 49 (26.6) | 50 (27.2) | 50 (27.2 | 35 (19.0) | 184 (100.0) |
| **France** | 17 (5.3) | 129 (40.1) | 76 (23.6) | 100 (31.1) | 322 (100.0) |
| **Italy** | 101 (84.2) | 5 (4.2) | 11 (9.2) | 3 (2.5) | 120 (100.0) |
| **Spain** | 0 (0.0) | 0 (0.0) | 26 (59.1) | 18 (40.9) | 44 (100.0) |
| **Portugal** | 4 (9.1) | 12 (27.3) | 13 (29.5) | 15 (34.1) | 44 (100.0) |
| **Luxemburg** | 32 (9.2) | 138 (39.7) | 89 (25.6) | 89 (25.6) | 348 (100.0) |
| **Gesamt** | **237 (17.0)** | **459 (32.8)** | **351 (25.1)** | **351 (25.1)** | **1,398** (100.0) |
