# Supplementary material for: COVID-19 and mental distress among health professionals in eight European countries during the third wave: a cross-sectional survey
Source: Sci Rep. 2024 Sep 12;14:21333. doi: 10.1038/s41598-024-72396-x (PMC11393084; doi:10.1038/s41598-024-72396-x)
Supplement: Supplementary file 2 — Supplementary Table 2. [file 41598_2024_72396_MOESM2_ESM.docx]

**Supplementary file 2: Results of the DASS-21 scales of 1,398 professionals in 8 different European countries**

Results of the DASS-21 depression scale of 1,398 professionals in 8 different European countries.

| **Country** | **Mean (SD)** | **Normal/mild,  n (%)** | **Moderate,  n (%)** | **Severe/  very severe  n (%)** |
| --- | --- | --- | --- | --- |
| Germany | 11.66 (10.41) | 152 (62.6) | 41 (16.9) | 50 (20.6) |
| Belgium | 14.84 (10.75) | 45 (48.4) | 16 (17.2) | 32 (34.4) |
| Switzerland | 8.45 (8.68) | 137 (74.5) | 32 (17.4) | 15 (8.2) |
| France | 16.52 (12.54) | 144 (44.7) | 63 (19.6) | 115 (35.7) |
| Italy | 9.67 (9.90) | 84 (70.0) | 19 (15.8) | 17 (14.2) |
| Spain | 6.55 (7.36) | 37 (84.1) | 3 (6.8) | 4 (9.1) |
| Portugal | 12.23 (11.38) | 28 (63.6) | 6 (13.6) | 10 (22.7) |
| Luxemburg | 12.59 (10.71) | 206 (59.2) | 59 (17.0) | 83 (23.9) |
| **Total** | **12.48 (11.09)** | **833 (59.6)** | **239 (17.1)** | **326 (23.3)** |

Results of the DASS anxiety scale of 1,398 professionals in 8 different European countries.

| **Country** | **Mean (SD)** | **Normal/mild,  n (%)** | **Moderate,  n (%)** | **Severe/  very severe  n (%)** |
| --- | --- | --- | --- | --- |
| Germany | 5.88 (6.95) | 185 (76.1) | 32 (13.2) | 26 (10.7) |
| Belgium | 9.89 (8.74) | 51 (54.8) | 22 (23.7) | 20 (21.5) |
| Switzerland | 4.35 (5.7) | 150 (81.5) | 22 (12.0) | 12 (6.5) |
| France | 10.88 (10.33) | 172 (53.4) | 51 (15.8) | 99 (30.7) |
| Italy | 5.65 (6.52) | 91 (75.8) | 20 (16.7) | 9 (7.5) |
| Spain | 8.41 (10.44) | 27 (61.4) | 10 (22.7) | 7 (15.9) |
| Portugal | 9.50 (9.71) | 26 (59.1) | 7 (15.9) | 11 (25.0) |
| Luxemburg | 8.36 (8.94) | 228 (65.5) | 49 (14.1) | 71 (20.4) |
| **Total** | **7.89 (8.79)** | **930 (66.5)** | **213 (15.2)** | **255 (18.2)** |

Results of the DASS-21 stress scale of 1,398 professionals in 8 different European countries.

| **Country** | **Mean (SD)** | **Normal/mild,  n (%)** | **Moderate,  n (%)** | **Severe/  very severe  n (%)** |
| --- | --- | --- | --- | --- |
| Germany | 15.84 (10.83) | 157 (64.6) | 36 (14.8) | 50 (20.6) |
| Belgium | 19.14 (11.24) | 49 (52.7) | 17 (18.3) | 27 (29.0) |
| Switzerland | 12.16 (9.36) | 143 (77.7) | 23 (12.5) | 18 (9.8) |
| France | 20.39 (11.67) | 146 (45.3) | 65 (20.2) | 111 (34.5) |
| Italy | 17.18 (10.93) | 70 (58.3) | 20 (16.7) | 30 (25.0) |
| Spain | 13.32 (10.74) | 33 (75.0) | 4 (9.1) | 7 (15.9) |
| Portugal | 20.00 (11.18) | 23 (52.3) | 4 (9.1) | 17 (38.6) |
| Luxemburg | 17.19 (11.23) | 208 (59.8) | 45 (12.9) | 95 (27.3) |
| **Total** | **17.13 (11.28)** | **829 (59.3)** | **214 (15.3)** | **355 (25.4)** |
